# Cases Occurring in Stanton Military General Hospital—Surg. John A. Lidell, U. S. V.—Gun-shot Wound of Head—Trephining—Death

**Published:** 1863-09

**Authors:** Wm. H. Gail

**Affiliations:** Medical Cadet, U. S. A.


ART. III.—*Cases occurring in Stanton Military General Hospital—Surg.* JOHN A. LIDELL, U. S. V.—*Gun-shot Wound of Head—Trephining—Death. Reported by* WM. H. GAIL, *Medical Cadet, U. S. A.*

Private Peter Hayes, Co. B, Ninth Pennsylvania Reserves, aged about 28 years, admitted to Stanton Hospital on the morning of December 15th, 1862; was wounded at the battle of Fredericsburg, Va., December 13th, 1862, in the head. Said he had no recollection of the receipt of the injury, and was unconscious for some time afterward. There was a scalp wound, such as would be inflicted by a bullet, over the posterior part of the parietal bone, on the left side of the head. On introducing the finger the wound was found to extend down to the bone, and the bone was depressed about one-fourth or one-sixth of an inch. There was another scalp wound about two inches anterior to the one just mentioned, through which he said a portion of a bullet had been extracted, before he came to this hospital. The last mentioned scalp wound was much smaller than the former, and the two were connected together underneath the scalp, as would be the case if the bullet had impinged upon the skull at the posterior wound, and glanced forward underneath the scalp. The patient complained of nothing but soreness in and about the scalp wound; he exhibited no symptom of compression or other lesion of the brain. He walked into the ward unassisted. The pulse and skin were good; the pupils were natural; he protruded his tongue well; there was no paralysis of any part of the body; he was perfectly conscious, and answered questions promptly and intelligently. He was ordered to remain quiet in bed; his hair was cropped close; ice was applied to his head, and the day passed without any alarming symptoms. During that night he had several convulsions, cataleptic in character, and brief in duration. At the morning visit of the 16th, he was found comatose, not very profoundly so, however, for he could be roused by speaking to him in a loud tone, and by introducing the finger into the wound, but he immediately sank off again into the state of stupor. His evacuations were passed involuntarily in bed He was subject to frequent twitchings of the muscles of the right side of his face. There was no disparity in the pupils, which were somewhat contracted; his limbs were not paralyzed; his pulse was preternaturally slow, and rather feeble; his skin cool and moist; (inflammation of the membranes of the brain, with effusion of its products, was the diagnosis.) Ice to head was continued, a blister was applied to nape of neck, and magnes. sulph. in oz. doses every four hours was given him, until free catharsis was produced. There was little change during the day.

December 17th. The coma deepened; the power of deglutition was lost, and the tip of the tongue turned to the left side of the mouth when protruded, notwithstanding the catharsis, blister, and the ice to the head. He had no control over his evacuations; the pupils were still symmetrical, and contracted readily with the stimulus of light; his pulse was slow and feeble; his skin cool and moist.

On the morning of the 18th, the patient being worse, exhibiting paralysis of the right arm, but not of the right leg, as a dernier resort, it was determined to apply the trephine and remove the depressed bone and fragments of internal table. Accordingly the patient was put under the influ ence of sulphuric ether, and the operation performed by Surgeon John A. Lidell, U. S. V. The crucial incision was made to expose the seat of fracture. One piece of bone, irregularly oval in shape, and rather more than one-half inch in diameter, was found to be depressed to the depth of from one-sixth to one-fourth of an inch. The crown of the trephine was applied to the anterior margin of the opening, in which situation it was observed that the pericranium was detached very easily. On removing the disk of bone excised by the trephine, the depressed portion was raised up, and easily removed with common dissecting forceps. Six fragments of the internal table were also removed; one of these of considerable size had perforated the dura-mater, making a rent about one-half an inch in length, and lacerating the substance of the brain; on withdrawing from that situation by the aid of forceps, there followed it a discharge of apparently disorganized brain substance.

After the operation the coma lightened considerably; the power of deglutition was restored, and he seemed sufficiently conscious to recognize his attendants, though he could not speak. In proof of partial recovery of consciousness, the nurse said he signalled to him when he wished to urinate. The improvement, however, was of short duration; on the afternoon of the 19th the coma again became more profound.

On the 20th, paralysis of the right side of the body became complete. After the operation frequent convulsive twitchings of the muscles of the right side of the face continued, but no general convulsive movements occurred. The symptoms increased in gravity continually; the patient became more and more exhausted, until mid-day on the 22d, nine days after receiving the injury, and four days subsequent to the operation, when he expired.

Autopsy 24 hours after death. Cadaver pale and emaciated; rigor mortis strong; on turning down the scalp, the track of the glancing ball from the posterior to the anterior wound was plainly seen; on dividing the dura-mater in the line of the saw, a thick and dark colored pus was discharged from the left side, to the amount of about one and one-half ounces; the dura-mater was seen to be congested, and on turning it up where it covers the convexity of the left cerebral hemisphere, its interior (parietal arachnoid) was seen to be lined over a great space, with a thick layer of false membrane; after removing the dura-mater, the greater part of the convex surface of the left cerebral hemisphere was seen to be covered over with a thick layer of yellowish gray colored plastic exudation, with brown spots, adhering to the visceral arachnoid; the surface of the right cerebral hemisphere presented a congested appearance; under the seat of injury to the cranium the substance of the brain was seen to be lacerated and prominent, and on slicing it carefully, it was found to have undergone red softening and disorganization to the depth of one and one-half inches, in that locality.

The substance of the cerebrum generally, including both right and left hemispheres, was congested; the puncta were unusually large and numerous, and on the cut surface of a section the open mouths of blood vessels were very distinct, so much so as to constitute the cribriform appearance. The ventricles contained a considerable quantity of clear serum, and a sero-sanguinolent effusion collected at the base of the skull. The visceral arachnoid about base of brain was opalescent.

